# ED Patients with Prolonged Complaints and Repeat ED Visits Have an Increased Risk of Depression

**DOI:** 10.5811/westjem.2016.7.30801

**Published:** 2016-08-08

**Authors:** Kristopher R. Brickman, Rajiv Bahl, Nathan F. Marcinkowski, Katelyn R. Ammons, Peter Akpunonu

**Affiliations:** The University of Toledo Medical Center, Department of Emergency Medicine, Toledo, Ohio

## Abstract

**Introduction:**

The objective of this study was to explore associations between presenting chief complaints of prolonged symptomatology, patient usage of the emergency department (ED), and underlying depression so that emergency physicians may better target patients for depression screening.

**Methods:**

A convenience sample of ED patients were administered the Beck Depression Inventory-II (BDI-II) to assess for depression. We correlated completed BDI-II surveys to patient information including demographics, pertinent history of present illness information, and past medical history.

**Results:**

Out of 425 participants screened, we identified complaints of two weeks or longer in 92 patients (22%). Of these patients, mild to severe depression was recognized in over half of the population (47), yet only nine patients reported a prior depression diagnosis. These 92 patients also visited the ED three times as frequently as those patients with more acute complaints (p<0.001). Finally, our study showed that patients with mild to severe depression had three times as many ED visits compared to patients with minimal or no depression (p<0.001).

**Conclusion:**

Patients with complaints of symptomatology two weeks or longer are more likely to have underlying depression when presenting to the ED. Patients with three or more ED visits within the past year also have a greater incidence of underlying depression. We found a strong correlation between complaints with symptomatology of two weeks or longer and multiple ED visits, in which underlying depression may have contributed to these patients’ ED visits.

## INTRODUCTION

This study was designed to explore the association between chief complaints of prolonged symptomatology, patient usage of the emergency department (ED), and underlying depression. We hypothesized that patients with complaints of symptomatology two weeks or longer present to the ED more frequently than those with more acute complaints, and that depression may be an underlying factor contributing to their medical symptoms. The ability to identify underlying depression in this population could provide an opportunity to better manage a patient’s overall care.

## METHODS

This prospective study was performed at The University of Toledo Medical Center Emergency Department (UTMC-ED), a Level I trauma center located in Toledo, Ohio. We collected data under the approval of the University of Toledo institutional review board (IRB): IRB#107866.

We used a convenience sample of patients presenting to the ED over a six-month period to select study participants. Subject enrollment occurred Monday through Sunday on a rotating shift to best ensure an even sampling of patients. Volunteering participants were provided an information sheet and waiver of consent. Subject exclusion criteria included those patients who were less than 18 years of age, non-English speaking, intoxicated, had critical illnesses, or who were otherwise medically unable to consent.

Participants were administered a Beck Depression Inventory-II (BDI-II), a self-reporting survey of 21 multiple-choice questions, to determine the presence and severity of depression symptoms as listed in the American Psychiatric Association’s Diagnostic and Statistical Manual of Mental Disorders 4^th^ Edition (DSM-IV). After completion of the BDI-II, subjects finished their participation in the study and no further communication relating to the study was conducted.^1^ Each question contained four to six weighted responses increasing in severity with answer ratings between 0–3. Completed surveys were hand scored by adding the corresponding response values chosen by the patient. We grouped patients by their level of depression via the BDI-II scores as follows: 0–13 having no or minimal depression, 14–19 with mild, 20–28 with moderate, and 29–63 with severe depression.

We reviewed electronic medical records of participating patients for the visit where the BDI-II was administered and any prior visits to the UTMC-ED within the preceding 12 months. Patient demographics, chief complaints, and past medical histories were recorded. Patient demographics were obtained from the patient’s medical record via a self-reported face sheet in triage given to all ED patients. For purposes of this study, we classified chief complaints as “chronic” symptomatology if the patient described the condition manifesting for two weeks or longer. The number of visits to the UTMC-ED one year prior to the visit when the BDI-II was administered was recorded.

We identified associations between categorical factors (e.g., medical history) and depression using chi-square or Fisher’s exact tests (for less common diagnoses). Mann-Whitney-Wilcoxon test was used for continuous factors (e.g., age, length of stay, ED visits). Because the continuous factors were not distributed normally, the median and interquartile ranges (IQR 25th to 75th percentile) are presented.

## RESULTS

We successfully enrolled 425 subjects who completed all study materials. Patients were grouped by their BDI-II scores, corresponding with their severity of depression symptoms. Of the 425 patients, 245 (58%) were classified with no or minimal depression; 62 patients (15%) mild depression; 85 patients (20%) moderate depression; and 33 (8%) severe depression (Figure). These findings are similar to previous studies calculating the prevalence of depression in the ED.^2^ For the remainder of this study, patients were grouped into two cohorts: patients with no or minimal depression (BDI-II score; 0–13) and patients with mild, moderate or severe depression (BDI-II score; 14–63) (Table 1).

Sample population demographics including sex, age and race are shown in Table 1. One hundred forty-nine patients (35%) were male and 276 (65%) were female. Females had a significantly higher percentage of depression than males (p=0.004). Two hundred twenty (52%) subjects indentified as Caucasian; 196 (46%) African American; and nine (2%) other. The median age was 32 years (IQR of 23 to 44). There was a significant association between self-reported depression in the ED and older age (p<0.001). Patients classified as having mild to severe depression had a median age of 35 years (IQR 26 to 48), while patients with no or minimal depression had a median age of 30 (IQR 22 to 41). These data are comparable to national statistics, which show the female gender and older age groups have an increased prevalence of depression.^3^

We divided the sample population by the classification of an acute versus chronic chief complaint during the visit when the BDI-II was completed. As shown in Table 2, of the 425 patients, 333 (78%) were classified as having a chief complaint with acute symptomatology and 92 (22%) had a chief complaint of chronic symptomatology. Patients presenting with a chronic chief complaint visited the UTMC-ED at three times the frequency of the acute patients (Table 2) (p<0.001).

Patients presenting with acute versus chronic chief complaints and number of ED visits were compared with their severity of depression determined via the subject’s BDI-II score (Table 3). We identified a significant association between more severe depression, a chronic chief complaint (p<0.05) and increased visits (p<0.001).

## DISCUSSION

Within EDs, physical symptoms are often misinterpreted as having an organic origin while mental illnesses, such as depression, may be the underlying cause or exacerbating the patient’s complaint. Studies estimate that 23–30% of ED patients have an underlying factor of depression and greater than 60% of patients with depression are unidentified.^4^,^5^,^6^ In a 2012 report, 79.7% of adults reported visiting the ED due to lack of access to other providers. 7 With a significant percentage of the U.S. population visiting the ED each year due to a lack of access to a primary source of healthcare, detecting depression in the ED is of utmost importance.^7^

Depression screening questionnaires are frequently used in the primary care setting. However, administering questionnaires to every patient who presents to the ED would be an impractical and costly process. The physician’s clinical judgment for the diagnosis of underlying depression is key, and then if needed, a questionnaire such as the Beck Depression Inventory-II (BDI-II) or Patient Health Questionnaire-9 (PHQ-9), can be used for confirmation. The BDI-II in our study classified 42% of the patients presenting to the UTMC-ED as having mild, moderate or severe depression. This figure is within range of other studies estimating the prevalence of depression using the BDI-II and the Hospital Anxiety and Depression Scales (HADS) questionnaires.^4^,^5^

EDs have become the safety net for many who seek treatment for chronic medical issues. These data signify that patients who present to the ED with chief complaints of symptomatology of two weeks or longer were more likely to have underlying depression compared to those with more acute complaints. In our study, the use of “two weeks or longer duration of symptoms” must be differentiated from a “chronic” complaint. Chronicity is defined by specific criteria for a medical illness, including the total duration of symptoms, the number of days symptoms are experienced and the number of days symptom free. To simply our criteria for clinical practice implementation, we used a patient self-reported duration of symptoms lasting two weeks or longer as a cutoff to be targeted for depression screening. Our data also exemplify that those patients who visited the ED three or more times in the preceding year have a significantly greater chance of having underlying depression compared to those with less frequent ED visits. Therefore, screening patients with frequent ED visits and prolonged symptomatology for depression may be beneficial in identifying and providing care for this mental health disorder.

Future studies are needed to access the appropriate channeling and coordination of care for patients identified with depression in the ED. Connecting these patients to appropriate mental health resources could be beneficial, but it is not known if these patients would be compliant with mental health follow up. Providing these patients with the resources to obtain follow-up care for their depression may be a suitable approach.

## LIMITATIONS

Limitations of this study are primarily based upon use of a convenience sampling of patients and an inventory to screen for depression. A convenience sampling of patients may limit the ability to generalize study findings to the population as a whole. Enrolling patients on a rotating shift and all days of the week was done to minimize this limitation. Like other self-reporting inventories, the BDI-II can have scores or emotions that are easily exaggerated or minimized by the person completing the survey.^8^ While this can be the case, overall, the BDI-II has strong correlations with the Hamilton Depression Rating Scale with a Pearson r of 0.71, suggesting good agreement and clinical correlation. Further, the BDI-II has high one-week retest reliability (Pearson r of 0.93), thus indicating it is not overly sensitive to daily variations in mood.^1^

## CONCLUSION

Based on this study, ED patients presenting with a chief complaint of symptomatology lasting two weeks or longer and those who have frequently presented to the ED in the past year have a significantly greater incidence of underlying depression. By targeting these high-risk ED patients for depression screening or follow up, emergency physicians may be able to better manage their care.

## Figures and Tables

**Figure:**
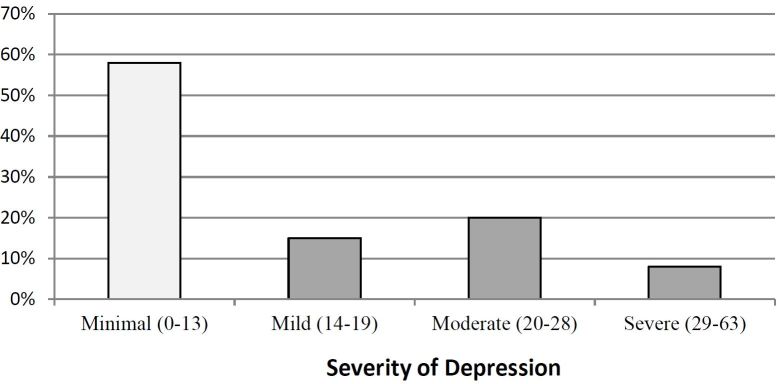
Patients classified by severity of depression (BDI-II score).

**Table 1:** Comparison of sample population demographics by severity of depression.

	Overall	Mild, moderate or severe depression	Minimal depression	P-value
Number of patients	425	180 (42%)	245 (58%)	--
Sex				0.004*
Female	276 (65%)	131 (73%)	145 (59%)	
Male	149 (35%)	48 (27%)	101 (41%)	
Age (years)	median 32 IQR 23 to 44	median 35 IQR 26 to 48	median 30 IQR 22 to 41	<0.001**
Race				0.38*
Caucasian	220 (52%)	98 (55%)	122 (51%)	
African American	196 (46%)	79 (45%)	117 (49%)	
Other	9 (2%)			

*IQR*, interquartile range;

* Chi-square p-value reported;

** Mann-Whitney-Wilcoxon test

**Table 2:** Number of emergency department (ED) visits by patients presenting with acute vs. chronic chief complaints.

	Acute complaint (<2wks)	Chronic complaint (>2wks)	P-value
Number of patients	333 (78%)	92 (22%)	--
ED visits	median 1 IQR 1 to 3	median 3 IQR 1 to 5	<0.001*

*ED*, emergency department; *IQR*, interquartile range;

* Chi-square p-value reported

**Table 3:** Chief complaints and number of emergency department (ED) visits by severity of depression.

	Overall	Mild, moderate or severe depression	Minimal depression	P-value
Number of patients	425	180 (42%)	245 (58%)	--
Chief complaint				0.05*
Acute (<2 weeks)	333 (78%)	133 (74%)	200 (82%)	
Chronic ( >2 weeks)	92 (22%)	47 (26%)	45 (18%)	
ED visits	median 2 IQR 1 to 4	median 3 IQR 1 to 6	median 1 IQR 1 to 3	<0.001*

*ED*, emergency department; *IQR*, interquartile range;

* Chi-square p-value reported
